# Aging and Weight-Ratio Perception

**DOI:** 10.1371/journal.pone.0047701

**Published:** 2012-10-24

**Authors:** Jessica S. Holmin, J. Farley Norman

**Affiliations:** 1 Department of Psychology, North Dakota State University, Fargo, North Dakota, United States of America; 2 Department of Psychology, Western Kentucky University, Bowling Green, Kentucky, United States of America; 3 Center for the Study of Lifespan Development, Western Kentucky University, Bowling Green, Kentucky, United States of America; McMaster University, Canada

## Abstract

Past research has provided evidence that older adults have more difficulty than younger adults in discriminating small differences in lifted weight (i.e., the difference threshold for older adults is higher than that of younger adults). Given this result, one might expect that older adults would demonstrate similar impairments in weight ratio perception (a suprathreshold judgment) compared to younger adults. The current experiment compared the abilities of younger and older adults to perceive weight ratios. On any given trial, participants lifted two objects in succession and were asked to provide an estimate of the objects’ weight ratio (the weight of the heavier object relative to the lighter). The results showed that while the older participants’ weight ratio estimates were as reliable as those of the younger participants, they were significantly less accurate: the older participants frequently perceived the weight ratios to be much higher than they actually were.

## Introduction

It is well known that increases in age adversely affect visual, tactile, and kinesthetic sensory functioning. For example, aging leads to significant deteriorations in visual acuity [1]–[3] and tactile acuity [4]–[7]; it is also accompanied by increases in weight-discrimination thresholds [8]. Interestingly, however, higher-level perceptual abilities are often not significantly affected by increases in age. Older adults can discriminate three-dimensional (3-D) object shape as well as younger adults for both the visual and haptic modalities [9]. Older adults can also haptically perceive 3-D surface shape [4] as well as younger adults.

Past research has demonstrated that good object shape perception does not necessarily require excellent visual acuity. Norman and colleagues [10] blurred their observers’ vision using 2.0-, 2.5-, and 3.0-diopter lenses; the use of these lenses reduced the observers’ acuity to 0.45, 0.67, and 0.92 LogMAR (log minimum angle of resolution), respectively. Even though blurring the observers’ vision produced severe deteriorations in acuity (e.g., a LogMAR acuity of 1.0 often represents legal blindness) [11], this manipulation did not appreciably affect the observers’ ability to discriminate 3-D object shape. Likewise, good haptic shape perception does not necessarily require excellent tactile acuity. The participants of Norman et al. [4] haptically explored 3-D surfaces and were required to estimate their shape; in addition, the participants’ tactile acuity was measured using a grating orientation discrimination task. In both of the experiments the older participants’ 3-D shape judgments were just as accurate as those of the younger participants despite the fact that the older participants’ grating orientation thresholds were 142.9 percent higher. Norman et al. [4] found that there was no significant relationship between the participants’ tactile acuity and their ability to haptically perceive 3-D surface shape. Given this pattern of past results (reductions in older adults’ sensory abilities do not necessarily impair their perceptual performance on everyday tasks involving objects), it is thus possible that while older adults’ ability to discriminate lifted object weight is reduced relative to younger adults [8], their ability to perceive weight ratios (a suprathreshold task) may be preserved.

Despite the fact that one cannot typically predict performance on suprathreshold perceptual tasks from the knowledge of a participant’s sensory (i.e., threshold) abilities, there are multiple (related) reasons why the perception of lifted object weight among older adults might be an exception. First of all, it is important to note that aging is associated with a loss of skeletal muscle mass, which leads to a reduction in strength (e.g., a 40 percent loss of muscle mass between the ages of 50 and 80 years results in a 50 percent decline in strength) [12]–[14]. Decades of research have revealed that the perception of an object’s weight is affected by the effort exerted by a participant during lifting [15], [16]. For example, studies have shown that participants perceive an object to be heavier after their muscles have become fatigued – because of the muscle fatigue, participants need to apply more effort (than usual) during lifting, which then causes an object to feel heavier [17]–[19]. In order to lift any particular object, older adults, in general, must apply more effort than younger adults because of their reduced muscle mass. Because of this increased application of effort, one might therefore expect that older adults’ perceptions of lifted object weight would be greater than those of younger adults. In addition to age-related changes in strength related to muscle loss, older adults utilize larger grip forces when lifting objects [20], [21]. It has been demonstrated that perceived object weight also depends upon grip strength, such that greater grip strengths are associated with increased perceptions of heaviness [22], [23]. Given the results of Flanagan and Wing [22], one might expect older adults’ perceptions of object weight to be greater than those of younger adults, because older adults grasp objects more strongly during lifting.

We know from our previous research [8] that older adults’ weight discrimination thresholds (i.e., minimum differences in weight needed to judge which of two weights is heavier) are more than 50 percent greater than those of younger adults. What we do not know, at present, is whether aging is associated with reduced performance on suprathreshold perceptual tasks involving judgments of object weight. As the previous review indicated, older adults can frequently perform well on tasks involving the perception of 3-D object shape despite impairments in visual and tactile acuity. If the perception of object weight follows a similar pattern, older adults may perform well on suprathreshold weight judgment tasks despite possessing impairments in weight discrimination. As we have seen, however, older adults’ abilities to perceive weight may be fundamentally different (than those involving the perception of object shape), because of the consequences of age-related muscle loss and changes in grip strength. The purpose of this experiment was to further investigate weight perception in younger and older adults to evaluate these possibilities.

## Materials and Methods

### Ethics Statement

The experiment was approved by the Western Kentucky University Human Subjects Review Board. The participants were students at Western Kentucky University or were recruited from the local community (Warren County, Kentucky), and all participants gave written consent prior to participation in the experiment.

#### Participants

Seventeen older adults (age range was 64 to 78 years, mean age was 68.9 years, SD = 4.6) and 17 younger adults (range was 18 to 31 years, mean age was 23.9, SD = 3.6) participated in the experiment. While changes in strength among older adults are proportionately similar for males and females, reductions in absolute strength tend to be greater for males (because of their higher baseline strength) [24]. Because of this, we included similar numbers of older males and females in the current study (8 older females and 9 older males). Two potential participants (one older and one younger) were excluded because they did not understand the task.

#### Apparatus

The order of presentation of the experimental stimuli was randomly determined for each participant by an Apple iMac computer. The participants’ weight-ratio judgments were entered into the computer for later analysis.

#### Experimental stimuli

The experimental stimuli were similar in size and shape to those used by Norman et al. [8]. The stimuli were small cylindrical bottles (4.9 cm diameter × 9.5 cm tall), which were filled with various amounts of #6 lead shot. The six object weights were 30, 55.9, 93.7, 145.5, 213.7, and 300 grams, the spacing of which was based on a cube-root transformation [25]. Two replicas for each weight were created, so that each weight could be paired with itself (and every other weight).

#### Procedure

There were 36 conditions (a result of each of the 6 stimuli being paired with every other stimulus and with itself) and three repetitions for each condition, creating a total of 108 trials. Every participant therefore made 108 judgments. The participants were visually separated from the stimuli and experimenter by an occluding barrier, through which participants placed their preferred arm. On any given trial, a randomly selected pair of stimuli was placed in front of the participants. The participants then lifted the objects one at a time and their task was to estimate a weight ratio (the ratio of the heavier object weight relative to the lighter; e.g., if the two weights on a particular trial were 30 and 300 grams, an accurate weight ratio judgment would be 10). During each trial, the participants could lift each object as many times as they wished up to a limit of 30 seconds. In addition, the participants were required to keep their elbows on the table at all times and lift with their forearms; they used their thumb and first two fingers to grasp the experimental stimuli.

In order to ensure that the participants clearly understood the task before beginning the experimental trials, they were shown objects clearly labeled “50 grams”, “100 grams”, “125 grams”, “150 grams”, and “400 grams”. The participants were then given several pairs of these objects and were told, for example, that 1) the 150 gram object weighed three times as much as the 50 gram object, 2) the weight of the 400 gram object was eight times that of the 50 gram object, 3) the weight of the 125 gram object was 25 percent heavier than the 100 gram object, etc. The participants were allowed to lift these sample objects (which had weights that were similar to, but different from those used in the actual experiment). The experimental trials did not begin until the participants understood that their task was to estimate a weight ratio (heavier object weight relative to the lighter object weight) for each of the object pairs.

**Figure 1:**
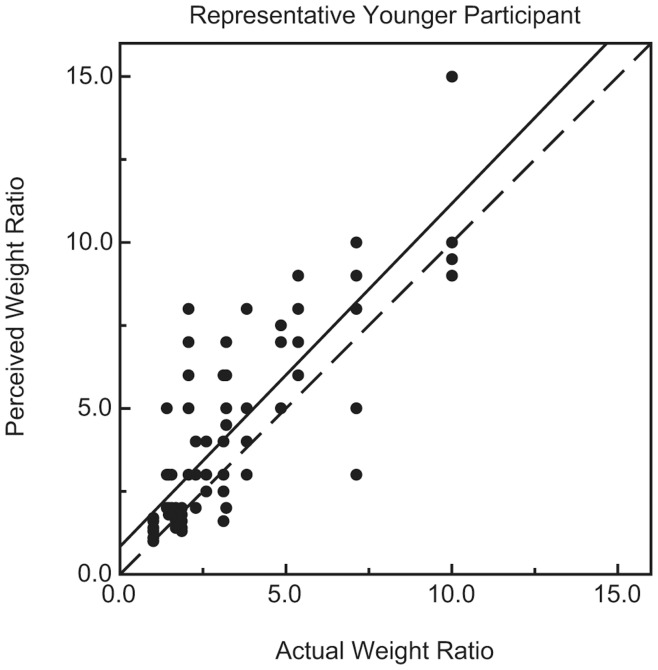
Individual results for a representative younger participant. The participant’s perceived weight ratios are plotted as a function of the actual weight ratios. The solid line indicates the best-fitting regression line. The dashed line represents accurate perceptual performance.

## Results

**Figure 2:**
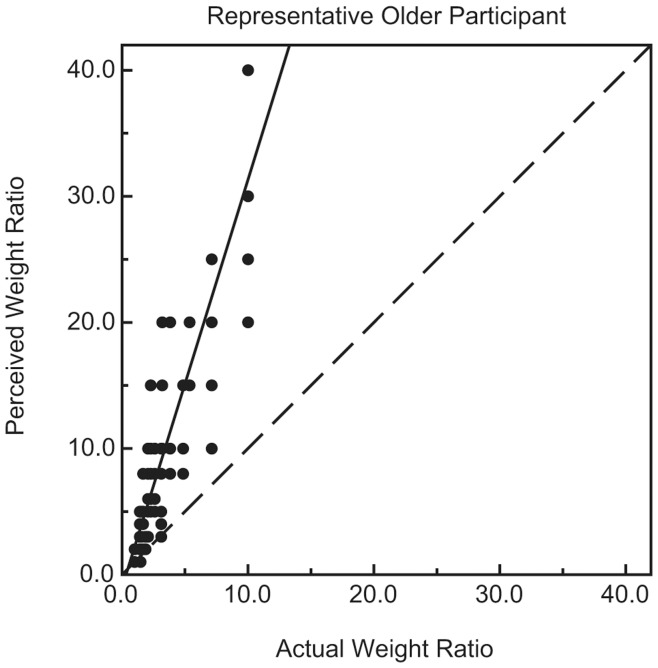
Individual results for a representative older participant. The participant’s perceived weight ratios are plotted as a function of the actual weight ratios. The solid line indicates the best-fitting regression line. The dashed line represents accurate perceptual performance.

**Figure 3:**
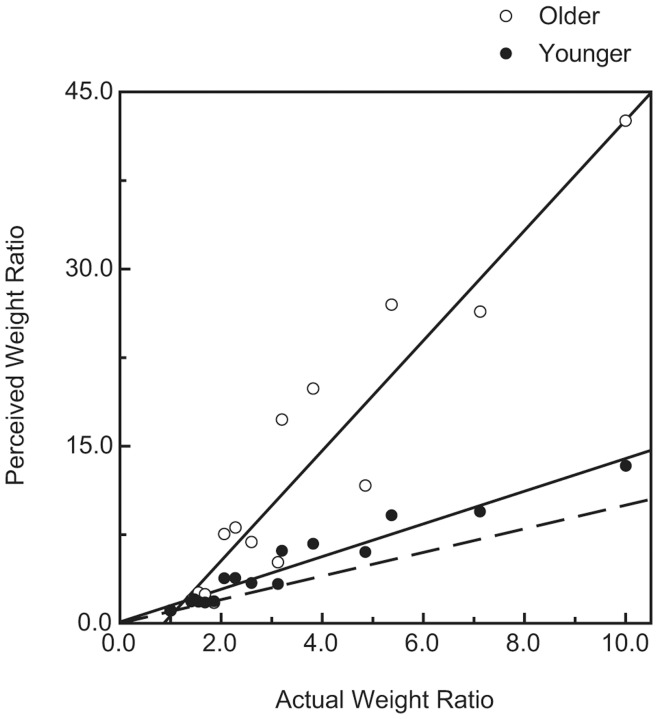
Average weight ratio estimates for all older and younger participants. The open circles indicate results for the older participants, while the filled circles indicate results for the younger participants. The solid lines indicate the best-fitting regression lines. The dashed line represents accurate perceptual performance.

**Figure 4:**
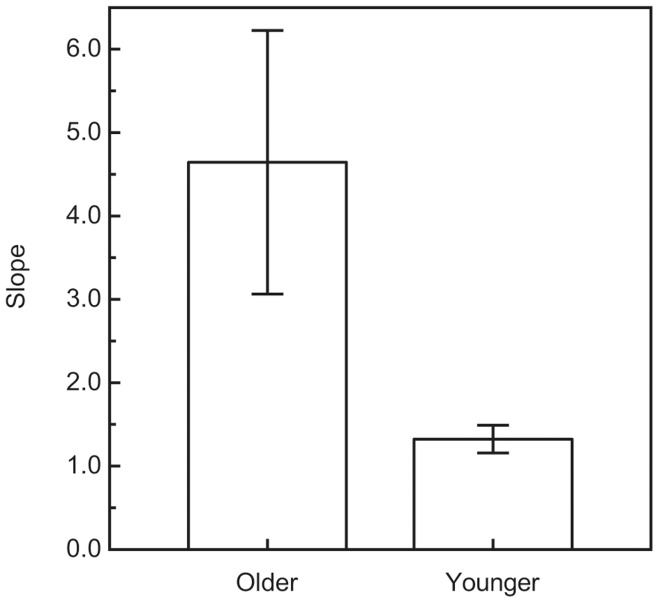
Mean slopes (of the best-fitting regression lines). The mean slopes are plotted separately for the younger and older groups of participants. The error bars indicate +/− one SE.

**Figure 5:**
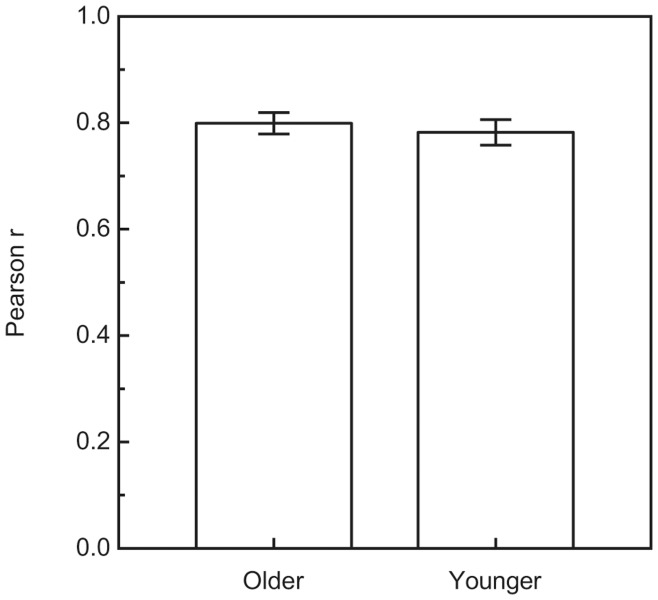
Mean Pearson r values. The r values are plotted separately for the older and younger groups of participants. The error bars indicate +/− one SE.

For every participant, the perceived weight ratios for the 108 stimulus pairs were plotted against the actual weight ratios. Figures 1 and 2 plot results for representative individual younger and older participants, while Figure 3 plots the average weight ratio estimates for all participants. Correlation coefficients (i.e., Pearson r values), along with the y-intercept and slope of the best-fitting regression line, were calculated for each individual participant. Because of greater variability among the older participants (e.g., in slope), a nonparametric test (Wilcoxon rank-sum test) [26], [27] was used to test for differences between the younger and older age groups. As can be seen in Figure 4, the mean slope (of the best-fitting regression line) of the older group was significantly higher than that of the younger group (W_x_ = 235, p = 0.031). In contrast, the magnitudes of the correlation coefficients for each group were not significantly different (see Figure 5; W_x_ = 283.5, p = 0.63). The difference in y-intercepts for the younger age group (M = −0.03, SD = 1.4) and the older age group (M = −3.6, SD = 10.6) was also not significant (W_x_ = 257.5, p = 0.17).

## Discussion

The results of the current experiment (e.g., Pearson r values) demonstrate that the weight-ratio judgments of the younger and older participants were equally reliable (see Figure 5). When there is little variability across repeated judgments (high reliability) for a participant, their data points cluster tightly about the regression line and the Pearson r value is high. Conversely, when there is high variability across repeated judgments (low reliability) for a participant, their data points fall farther (on average) from the regression line and the Pearson r value is low. In our experiment, the average Pearson r values were 0.80 and 0.78 for the older and younger participants, respectively. The weight-ratio judgments for individual older participants were no more variable across repeated trials than the judgments of younger participants (e.g., this can also be seen by examining Figures 1 and 2, which plot individual results for representative younger and older participants). It is important to note that in our experiment, more than 60 percent (0.78^2^ = 0.61) of the variance in both the younger and older participants’ weight ratio estimates could be accounted for by variations in the actual object weight ratios.

While the weight-ratio judgments of our older participants in the current study were just as reliable as those of the younger participants, they were not as accurate. Accurate judgments would produce regression lines with a slope of 1.0 and a y-intercept of zero. Notice from Figures 3 and 4 that while the slopes of regression lines obtained for the younger participants were relatively close to 1.0 (the mean slope was 1.32), the slopes of regression lines obtained for the older participants were much higher (mean slope was 4.64). The results for our younger participants are similar to those of past research. Baker and Dudek [28] also asked their participants to judge weight ratios. When their participants’ perceived weight ratios are plotted as a function of actual weight ratios, the data (see their Table 6) [28] produce a slope of 1.56. Like Baker and Dudek, we found that our younger participants’ judgments of weight ratios were not perfectly accurate (slopes somewhat higher than 1.0). Nevertheless, the judgments of our older participants were much more inaccurate than those of our younger participants. The haptic perception of surface shape follows a quite different pattern. Norman et al. [4] found that when older participants haptically estimate 3-D surface shape, their judgments were just as accurate as younger adults–there was no effect of age.

Given the results of current and past research, it seems clear that there is no overall or general decline in tactile, haptic, or lifted weight perception abilities as people age. The effects of aging are task dependent, so that some tasks produce marked age differences [4]–[8], while for other tasks older adults perform as well as younger adults [4], [9]; similar task-specific effects of age have been found by Billino et al. [29] and Norman and colleagues [30], [31] with respect to the visual perception of motion.

Historically, neurological testing of medical patients included an evaluation of patients’ weight discrimination abilities [32]. Impairments in weight perception were found to occur as a result of brain injury [33]–[36] to the parietal lobe. Within the parietal lobe, the supramarginal gyrus and parietal operculum appear to be especially important for judgments of object weight [37], [38]. In this context, it is important to note that aging affects the parietal lobe more severely than other lobes of the brain heavily involved in basic perceptual abilities (i.e., more neuronal cell death and cortical atrophy in the parietal as compared to the temporal and occipital lobes) [39]. Thus, relatively simple tests of weight discrimination and weight-ratio perception, such as those used by Norman et al. [8] and in the current study, may serve as useful clinical indicators of the severity of age effects upon the brain.
